# Co-Expression of Wild-Type P2X7R with Gln460Arg Variant Alters Receptor Function

**DOI:** 10.1371/journal.pone.0151862

**Published:** 2016-03-17

**Authors:** Fernando Aprile-Garcia, Michael W. Metzger, Marcelo Paez-Pereda, Herbert Stadler, Matías Acuña, Ana C. Liberman, Sergio A. Senin, Juan Gerez, Esteban Hoijman, Damian Refojo, Mišo Mitkovski, Markus Panhuysen, Walter Stühmer, Florian Holsboer, Jan M. Deussing, Eduardo Arzt

**Affiliations:** 1 Instituto de Investigación en Biomedicina de Buenos Aires (IBioBA)-CONICET- Partner Institute of the Max Planck Society, Buenos Aires, Argentina; 2 Departamento de Fisiología y Biología Molecular y Celular, Facultad de Ciencias Exactas y Naturales, Universidad de Buenos Aires, Buenos Aires, Argentina; 3 Max Planck Institute of Psychiatry, 80804, Munich, Germany; 4 Affectis Pharmaceuticals, 44227, Dortmund, Germany; 5 Centro de Microscopías Avanzadas, Facultad de Ciencias Exactas y Naturales, Universidad de Buenos Aires, Buenos Aires, Argentina; 6 Max Planck Institute of Experimental Medicine, 37075, Göttingen, Germany; 7 HMNC Brain Health, Munich, Germany; Albert Einstein College of Medicine, UNITED STATES

## Abstract

The P2X7 receptor is a member of the P2X family of ligand-gated ion channels. A single-nucleotide polymorphism leading to a glutamine (Gln) by arginine (Arg) substitution at codon 460 of the purinergic P2X7 receptor (P2X7R) has been associated with mood disorders. No change in function (loss or gain) has been described for this SNP so far. Here we show that although the P2X7R-Gln460Arg variant per se is not compromised in its function, co-expression of wild-type P2X7R with P2X7R-Gln460Arg impairs receptor function with respect to calcium influx, channel currents and intracellular signaling in vitro. Moreover, co-immunoprecipitation and FRET studies show that the P2X7R-Gln460Arg variant physically interacts with P2X7R-WT. Specific silencing of either the normal or polymorphic variant rescues the heterozygous loss of function phenotype and restores normal function. The described loss of function due to co-expression, unique for mutations in the *P2RX7* gene so far, explains the mechanism by which the P2X7R-Gln460Arg variant affects the normal function of the channel and may represent a mechanism of action for other mutations.

## Introduction

The purinergic P2X7 receptor (P2X7R) is a member of the P2X family of ligand-gated ion channels [1], which responds to ATP as the endogenous ligand [2]. Although its structure is similar to other members of the P2X receptor family that have two transmembrane domains and an extracellular loop as well as intracellular N- and C-termini, the P2X7R has a much larger intracellular C-terminal domain, which may be responsible for the additional functions of this receptor. P2X7R has been shown to form homomeric trimers and hexamers and might also form heteromultimers with P2X4 [3–5]. Unlike other family members, homotrimers are the predominant P2X7R form in vivo [6].

Short stimulation of P2X7R with extracellular ATP, or the more potent agonist 2′,3′-*O*-(benzoyl-4-benzoyl)-ATP (BzATP), activates Ca^2+^ influx. Prolonged or repeated exposure to the ligand opens a non-selective cation channel with considerable calcium permeability and induces the formation of a cytolytic pore permeable to large hydrophilic molecules such as ethidium bromide, eventually leading to cell death [7, 8].

The biochemical function of P2X7R is not restricted to opening calcium channels and membrane pores. The P2X7R was shown to mediate the activation of extracellular signal-regulated kinases (ERK 1/2) in rat primary astrocytes and astrocytoma cells linking ATP-induced P2X7R stimulation to ERK 1/2 activation which could lead to a better understanding of the pathophysiological roles this receptor may play in brain diseases [9, 10].

The P2X7R is predominantly expressed in immune, endothelia, and epithelia cells regulating various aspects of immune function, including expression and secretion of cytokines and inflammatory mediators [11–15]. It also plays an important role in inducing apoptosis, depending on the intensity of receptor stimulation [16].

In recent years, the role of P2X7R in the central nervous system has attracted considerable attention [17]. P2X7R is expressed throughout the central nervous system, in all glial lineages and in certain populations of neurons, this latter with low expression levels (reviewed in [18]). Different studies using pharmacological approaches have demonstrated a role of P2X7R in the regulation of diverse neural functions such as neurotransmitter release, and also in microglia and astroglial activation [11, 19–24].

Due to the central role of P2X7R in different biological processes, association studies of mutations in its nucleotide sequence and various different diseases were conducted revealing susceptibility to leukemia, tuberculosis and osteoporosis conferred by receptor gene polymorphisms [25–27]. Several variants causing loss-of-function of the P2X7R have been identified, such as polymorphism 1729T>A (Ile568Asn) [28], 946G>A (Arg307Gln) [29], 1352T>C (Pro451Leu) [30], and 1513A>C (Glu496Ala) [31]. A polymorphism located in the extracellular portion of the receptor, the 489C>T (His155Tyr) variant, showed a significantly increased calcium influx, being a gain-of-function polymorphism of the P2X7R [25].

A particularly strong case for an association of P2RX7 gene mutations and disease susceptibility was found for major depression and bipolar disorder [32–37]. In these studies, a heterozygote disadvantage model was the most suitable mode of inheritance, which is consistent with the multimeric nature of P2X7R channels [34]. The 1405 A>G polymorphism that results in the aminoacid change Gln460Arg (P2X7R-Gln460Arg) in the intracellular domain of the channel is more frequent in patients with both kinds of mood disorder when they are heterozygotes [34]. Notably, some other studies did not detect significant associations of the Gln460Arg polymorphism with mood disorder [38–41]. The Gln460Arg polymorphism, located in the long cytoplasmic tail of the receptor, has shown not to have an effect on P2X7R function when transfected in P2X7R-negative HEK293 cells [25, 42].

The functional features of the single-point mutation P2X7R-Gln460Arg in relation to the normal channel have not yet been studied. Thus, the aim of this work is to establish whether there is an altered function of the P2X7R bearing both subunits, P2X7R-WT and P2X7R-Gln460Arg. To accomplish this, we employed human cell lines ectopically expressing P2X7R variants and analyzed calcium intake, channel currents and intracellular signaling. We show that P2X7R-WT and P2X7R-Gln460Arg interact, leading to a diminished function and compromised transduction of the intracellular signaling.

## Materials and Methods

### Cell culture and generation of stable clones

HEK293 human cells were kindly provided by Dr Francesco Di Virgilio (University of Ferrara, Italy) [25].

Cells were cultured in DMEM (FCS 10%, L-glutamine 4 mM, Hepes 10mM, NaHCO_3_ 2.2g/liter, penicillin 100 U/ml, streptomycin 100 mg/ml, glucose 4.5 mg/ml) and kept at 37°C in a humidified 5% CO_2_ incubator.

Clones stably expressing human P2X7R-WT and P2X7R-Gln460Arg were generated Cells were maintained in selection medium G-418 (Life Technologies, Carlsbad, California) 800 μg/ml or Zeocin (Life Technologies) 100 μg/ml to obtain resistant clones.

### Plasmid constructs

The full-length P2X7R cDNA was amplified by PCR from human hippocampus cDNA using primers: forward: 5'-CAC-CAT-GCC-GGC-CTG-CTG-CAG-CTG-CAG-TGA-TGT-TTT-3' and reverse: 5'-GTA-AGG-ACT-CTT-GAA-GCC-ACT-GTA-CTG-CCC-TTC-ACT-3'.

The single nucleotide polymorphism (SNP) variant of P2X7R was constructed using the GeneTailor Site-Directed Mutagenesis System (Life Technologies) and primers: Gln460Arg forward: 5’-GGA-CAA-CCA-GAG-GAG-ATA-C**G**G-CTG-CTT-AGA-3’; Gln460Arg reverse: 5’-GTA-TCT-CCT-CTG-GTT-GTC-CAG-GAA-TCG-GG-3’ (nucleotide exchanges compared to the template are in boldface type and underlined). The hP2X7R-WT and hP2X7R-Gln460Arg cDNAs were introduced into the pcDNA3 and/or pcDNA3-Zeo expression vector. For generation of STREP-hP2X7R-WT and HIS-hP2X7R-Gln460Arg the cDNAs were introduced into the pEXPR-IBA7 STREP-tag plasmid or the pEXPR-IBA43 6xHistidine-tag plasmid. For generation of Cerulean and Venus constructs hP2X7R-WT or hP2X7R-Gln460Arg were ligated in frame into the polylinker region of pEYFP-N1 (Clontech, Mountain View, California). Afterwards, EYFP was replaced by either Cerulean or Venus fluorescent variants amplified by PCR. The nucleotide sequences of all constructs were confirmed by DNA sequencing.

### Real-Time PCR (qRT-PCR)

For specific quantification of P2X7R-WT and -Gln460Arg transcripts by quantitative Real-Time PCR the following primers were used: P2X7R-WT forward primer: 5’-GGA-CAA-CCA-GAG-GAG-ATA-CA-3’; P2X7R-WT reverse primer: 5’-TGG-TAG-AGC-AGG-AGG-AAC-TG-3’ (length of PCR product: 230 bp). P2X7R-Gln460Arg forward primer: 5’-GAA-CCA-GCA-GCT-ACT-AGG-GAG-AAG-3’; P2X7R-Gln460Arg reverse primer 5’-GAG-TCG-CCT-CCT-TTC-TAA-GCA-GCC-3’ (length of PCR product: 167 bp). Amplifications were performed in 2.5 mM MgCl_2_ and DMSO 2% for P2X7R and 2.5 mM MgCl2 for actin primers. 1:30000 SYBR Green (Life Technologies) was used as fluorescent dye. The quantification standard curves for P2X7R-WT mRNA and for P2X7R-Gln460Arg mRNA were normalized to actin mRNA. For quantification of P2X family members the following primers were used: hP2RX1-for 5´-GGC-CCT-TGA-GTT-TCA-CAG-AG-3´, hP2RX1-rev 5´-GTC-CTG-GTC-TAC-GTC-ATC-GG-3´, hP2RX2-for 5´-CCC-TTG-ACC-TTG-GTG-ATG-AT-3´, hP2RX2-rev 5´-ACT-ACG-AGA-CGC-CCA-AGG-T-3´, hP2RX3-for 5´-CAC-TGC-CAT-TTT-CCA-TTT-TG-3´, hP2RX3-rev 5´-AAT-ACT-CCT-TCA-CCC-GGC-TC-3´, hP2RX4-for 5´-CCC-TGT-GTC-TGG-TTC-ATG-GT-3´, hP2RX4-rev 5´-GTG-CAA-CTG-CTC-ATC-CTG-G-3´, hP2RX5-for 5´-CAG-GTC-GCA-GAA-GAA-AGC-A-3´, hP2RX5-rev 5´-GAT-ATT-ACC-GAG-ACG-CAG-CC-3´, hP2RX6-for 5´-ACT-TCG-TGA-AGC-CAC-CTC-AG-3´, hP2RX6-rev 5´-CCC-TAG-GAG-GCA-AGT-CTC-AA-3´, hP2RX7-for3 5´-ATG-TCA-AGG-GCC-AAG-AAG-TC-3´, hP2RX7-rev3 5´-AGG-AAT-CGG-GGG-TGT-GTC-3´, hRPL19-for 5´-GGA-TTC-TCA-TGG-AAC-ACA-T-3´, hRPL19-rev 5´-CTG-GTC-AGC-CAG-GAG-CTT-3´.

### Calcium imaging

All experiments were performed at room temperature (20–24°C). Cells in 40 mm Petri dishes were loaded for 45 min in darkness with Fluo-4 AM 6 μM (Molecular Probes) and Pluronic F-127 0.14% (Molecular Probes) in a Ca^2+^-buffer with low Ca^2+^ concentration (125 mM NaCl, 5 mM KCl, 0.4 mM CaCl_2_, 1 mM MgSO_4_, 5 mM NaHCO_3_, 1 mM Na_2_HPO_4_, 10 mM glucose, 20 mM Hepes pH 7.4), and then placed on the stage of a fluorescence BX-FLA Olympus microscope. The cells were illuminated with a USH-I 02DH mercury lamp (USHIO) and imaged using 40X water immersion objective and a cooled CCD Quantix camera (Photometrix). Exposure times were between 100 and 300 ms, and frames were taken every 5 s for the first minute and every 10 s intervals afterwards. Images were acquired with the Axon Imaging Workbench 2.1 program and analyzed with Image J 1.29v (NIH). Calcium imaging data are presented as ΔF/F_o_, where F_o_ is the resting fluorescence (before stimulation) and ΔF is the peak change in fluorescence from resting levels.

### Patch Clamp analysis

Whole cell recordings were obtained using an EPC9 patch clamp amplifier and PULSE acquisition programs (HEKA Elektronik). The holding potential was set to –70 mV. The extracellular solution (buffer with low Ca^2+^ concentration and no Mg^2+^) contained (in mM) 150 NaCl, 2.5 KCl, 0.3 CaCl_2_, 10 glucose and 10 HEPES, pH 7.4 (NaOH). The intracellular solution contained (in mM) 130 KCl, 10 CsF, 10 NaF, 10 EGTA and 10 HEPES, pH 7.4 (KOH). A computer-driven perfusion pipette (ALA-VM8; ALA Scientific Instruments) switched the extracellular medium during the recordings from normal extracellular solution to one containing, in addition, 50 μM BzATP for 10 s and back. Series resistance was compensated up to 80%. The current responses were normalized with respect to cell capacitance obtained from slow capacitance compensation.

### Coimmunoprecipitation and Western blotting

HEK293 clones containing STREP/HIS tagged hP2X7R variants were lysed on ice with modified RIPA buffer (Triton X-100 1% and SDS 0.1%), and immunoprecipitated with either anti-STREP tag (IBA, Göttingen, Germany) or anti-HIS tag (Qiagen, Hilden, Germany). Afterwards, IP extracts were analyzed by SDS-PAGE and immunoblotted with the same antibodies.

For WB on crude extracts, cellular lysates were analyzed by SDS-PAGE followed by immunoblotting using antibodies for pERK 1/2 (1:500; Santa Cruz, Dallas, Texas), ERK 1/2 (1:1000; Cell Signaling Technology, Danvers, Massachusetts), P2X7R c-terminal domain (1:1000, Alomone Labs, Jerusalem, Israel) and tubulin (1:10000, Abcam, Cambridge, Massachusetts). Cellular lysates were obtained from three independent experiments; one representative immunoblot is shown. Signals were quantified by densitometry using ImageJ software (National Institutes of Health).

### FRET microscopy

HEK293 cells were grown on 25mm diameter cover glasses placed on 6-well plates, and transfected at 60% confluency with Lipofectamine 2000 (Invitrogen) according to manufacturer’s instructions. Fluorescence observations were performed at 37°C on an IX81 –inverted microscope by using a 60X, 1.35 numerical aperture, oil-immersion objective (Olympus, Center Valley, Pennsylvania). Confocal images were acquired using an Olympus Fluoview FV1000 microscope, with a Multi Argon-Ion laser– 30 milliwats (457, 488, 515 nanometers). The three different settings used for the analysis of FRET with the Cerulean—Venus pair were (i) FRET: Ex 458 nm/Em 530–630 nm, (ii) Donor: Ex 458 nm/Em 466–494 nm, (iii) Acceptor: Ex 515 nm/Em 530–630 nm. Image acquisition was performed by using FV10-ASW software (Olympus). ImageJ version 1.43 with the PixFRET plugin was used for quantification of generated images of sensitized-emission FRET by computing on a pixel-by-pixel basis [43]. Donor and acceptor spectral bleed-throughs in the FRET settings were determined on cells expressing the donor (P2X7R-Cerulean) or the acceptor (P2X7R-Venus) alone by calculating the intensity (I) ratios in the appropriate settings after background substraction:

$$SBT_{Donor}\;=\;\frac{I_{FRET}}{I_{Donor}},$$

when the donor alone is expressed,

$$SBT_{Acceptor}\;=\;\frac{I_{FRET}}{I_{Acceptor}},$$

when the acceptor alone is expressed.

FRET measured in co-expressing cells was then corrected for spectral bleed-throughs and normalized (NFRET) for expression levels according to the following formula:

$$NFRET\;=\;\frac{I_{FRET}\;-\;SBT_{Donor}\times \;I_{Donor}-SBT_{Acceptor}\;\times \;I_{Acceptor}}{\sqrt{I_{Donor}\;\times \;I_{Acceptor}}}\;\times 100$$

Normalizing FRET values to the square root of the product of the donor and acceptor fluorescence intensities controlled for large variations in the expression levels of each fluorophore between different cells and provided a measure of FRET that is readily comparable between different samples [44].

Under the same conditions, HEK293 cells either untransfected or expressing P2X7R channels without fluorescent tags yielded very low endogenous autofluorescence. As a negative control, HEK293 cells were transfected with Cerulean and Venus fused to two noninteracting membrane proteins, P2X7R-WT and the NGF receptor subunit Trk-A, respectively. Both fluorescently tagged proteins were expressed at the membrane level, but showed no FRET signal.

### siRNA transfection

siRNAs designed to selectively silence either P2X7R-WT or P2X7-Gln460Arg were as follows: siRNA against P2X7R-WT: 5’-AAC-CAG-AGG-AGA-UAC-AGA-UTT-3’ and siRNA against P2X7R-Gln460Arg: 5’-AAC-CAG-AGG-AGA-UAC-GGA-UTT-3’. A scramble siRNA was used as a control: 5’-CUU-ACG-CUG-AGU-ACU-UCG-ATT-3’. Cells were transfected for 6 h with 100 nM siRNA using Lipofectamine 2000 (Life Technologies) on OPTIMEM (Life Technologies). Then, cells were washed and kept in culture with DMEM 10% FCS for 72 h, before the corresponding assay was performed.

### Statistical analysis

Data and statistical analysis were performed with the computer program GraphPad Prism 5.0. All results are shown as means ± standard error of the mean (s.e.m.). Calcium Imaging data were analyzed by repeated measures ANOVA. Patch Clamp and real-time PCR data was analyzed by one-way ANOVA with Scheffé’s test. Significance was accepted at *P* = 0.05.

## Results

### P2X7R-WT and P2X7R-Gln460Arg expressed in P2X7R negative HEK293 cells have equal functionality

To study the action of the P2X7R-Gln460Arg on receptor function we used a HEK293 cell line that endogenously does not express P2X7R, to generate stable clones expressing either human wild-type P2X7R (hP2X7R-WT) or the Gln460Arg receptor variant (hP2X7R-Gln460Arg), respectively. The absence of endogenous expression of the P2X7R gene in the parental HEK293 cell line and P2X7R-WT or P2X7R-Gln460Arg expression in the stably expressing clones was confirmed by qRT-PCR and Western blot (WB; S1A–S1C Fig).

**Fig 1:**
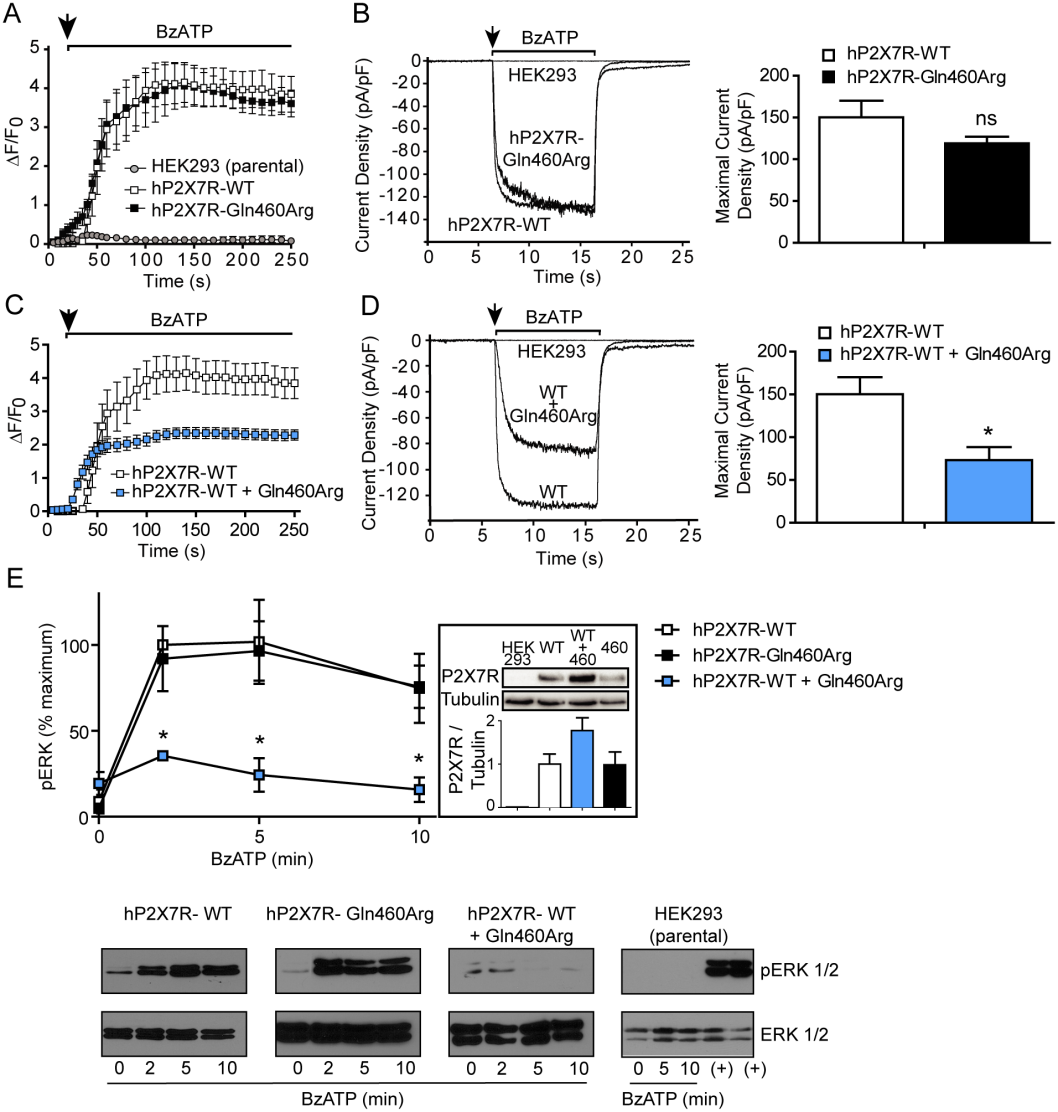
Co-expression of hP2X7R-Gln460Arg with hP2X7R-WT diminishes normal receptor function. (A) Increase of intracellular calcium of stably transfected HEK293 cells was measured following BzATP application (50 μM) (repeated measures ANOVA, *P* < 0.01 hP2X7R-WT and hP2X7R-Gln460Arg versus HEK293; n = 4). For each cell line, nine individual clones were analyzed (B) Left: representative whole-cell measurements out of four independent experiments by whole-cell patch clamp analysis. Right: Quantification of inward currents elicited by BzATP (One-way ANOVA with Scheffé’s test, ns = non-significant versus hP2X7R-WT; n = 4) (C) Increase of intracellular calcium of HEK293 cells stably transfected with hP2X7R-WT (9 clones) and stably double transfected with hP2X7R-WT + hP2X7R-Gln460Arg (10 clones) was measured (repeated measures ANOVA, *P* < 0.01 hP2X7R-WT + Gln460Arg versus hP2X7R-WT; n = 4). (D) Left: representative whole-cell measurements by whole-cell patch clamp analysis. Right: Quantification of inward currents elicited by BzATP (One-way ANOVA with Scheffé’s test, *P < 0.05 versus hP2X7R-WT; n = 4) (E) BzATP (50 μM)-induced activation of p-ERK 1/2 in HEK293 cells expressing P2X7R variants. Each value of pERK1/2 was normalized to total ERK1/2. Results are expressed as the percentage of maximum pERK1/2 obtained at 2 minutes of stimulation in hP2X7R-WT cells ± s.e.m. from 3 independent experiments. One-way ANOVA, * P < 0.05 versus hP2X7R-WT and versus hP2X7R-Gln460Arg at the same time points. Bottom panels show WBs of pERK1/2 and total ERK1/2 from a representative experiment. (+): Fetal calf serum 10% treatment for 10 min, positive control for p-ERK 1/2 activation. Inset: Quantification and representative example showing WB detection of hP2X7R variants in parental HEK293 cells and analyzed stable clones.

In both types of stable HEK293 clones BzATP induced a similar rapid increase of intracellular calcium and of currents assessed by whole-cell patch clamp analysis, whereas no response was observed in non-transfected parental HEK293 cells (Fig 1A and 1B). These results are in agreement with previous publications [25, 42] where the P2X7R-Gln460Arg variant showed no differences to P2X7R-WT on parental HEK293 cells.

### Co-expression of P2X7R-Gln460Arg together with P2X7R-WT diminishes P2X7R normal function in P2X7R negative HEK293 cells

In order to detect the outcome of co-expression of P2X7R-WT and P2X7R-Gln460Arg, as it would be the case of a heterozygous situation, one clone of the first round of transfection was stably transfected with pcDNA3-P2X7R-Gln460Arg expression vector and ten clones co-expressing P2X7R-WT and P2X7R-Gln460Arg were obtained.

qRT-PCR and WB confirmed the mRNA and protein expression levels of P2X7R-WT and P2X7R-Gln460Arg in the stable double clones (S1A–S1C Fig). The total amount of P2X7R protein in most of the stable double clones is about the double that in the single clones (S1C Fig).

The co-expression of both P2X7R-WT receptor and the P2X7R-Gln460Arg variant caused a significant reduction of normal receptor function in all the ten co-expressing clones analyzed (Fig 1C). These results were further confirmed by patch clamp analysis (Fig 1D) showing reduced inward currents compared to the activation of a P2X7R-WT clone in response to BzATP application.

### P2X7R-dependent ERK 1/2 activation is affected in P2X7R-WT and P2X7R-Gln460Arg stable double clones

In order to determine if the different P2X7R activation levels have an impact on downstream intracellular signaling pathways, we examined BzATP-induced ERK 1/2 phosphorylation in stable HEK293 clones. In response to BzATP, cells stably expressing either P2X7R-WT or P2X7R-Gln460Arg showed a robust time-dependent ERK 1/2 phosphorylation (Fig 1E). In contrast, there was a diminished response to BzATP in P2X7R-WT and P2X7R-Gln460Arg co-expressing HEK293 cells (Fig 1E). As in the case of calcium assays, a complete absence of ERK 1/2 response was observed in non-transfected parental HEK293 cells, further confirming that BzATP effects are elicited through P2X7 receptors in these cells.

The blunted calcium intake and ERK 1/2 phosphorylation in response to BzATP observed on cells co-expressing P2X7R-WT and P2X7R-Gln460Arg provide evidence for a role of co-expression on altered receptor function.

### P2X7R-Gln460Arg interacts with P2X7R-WT at the cell membrane

**Fig 2:**
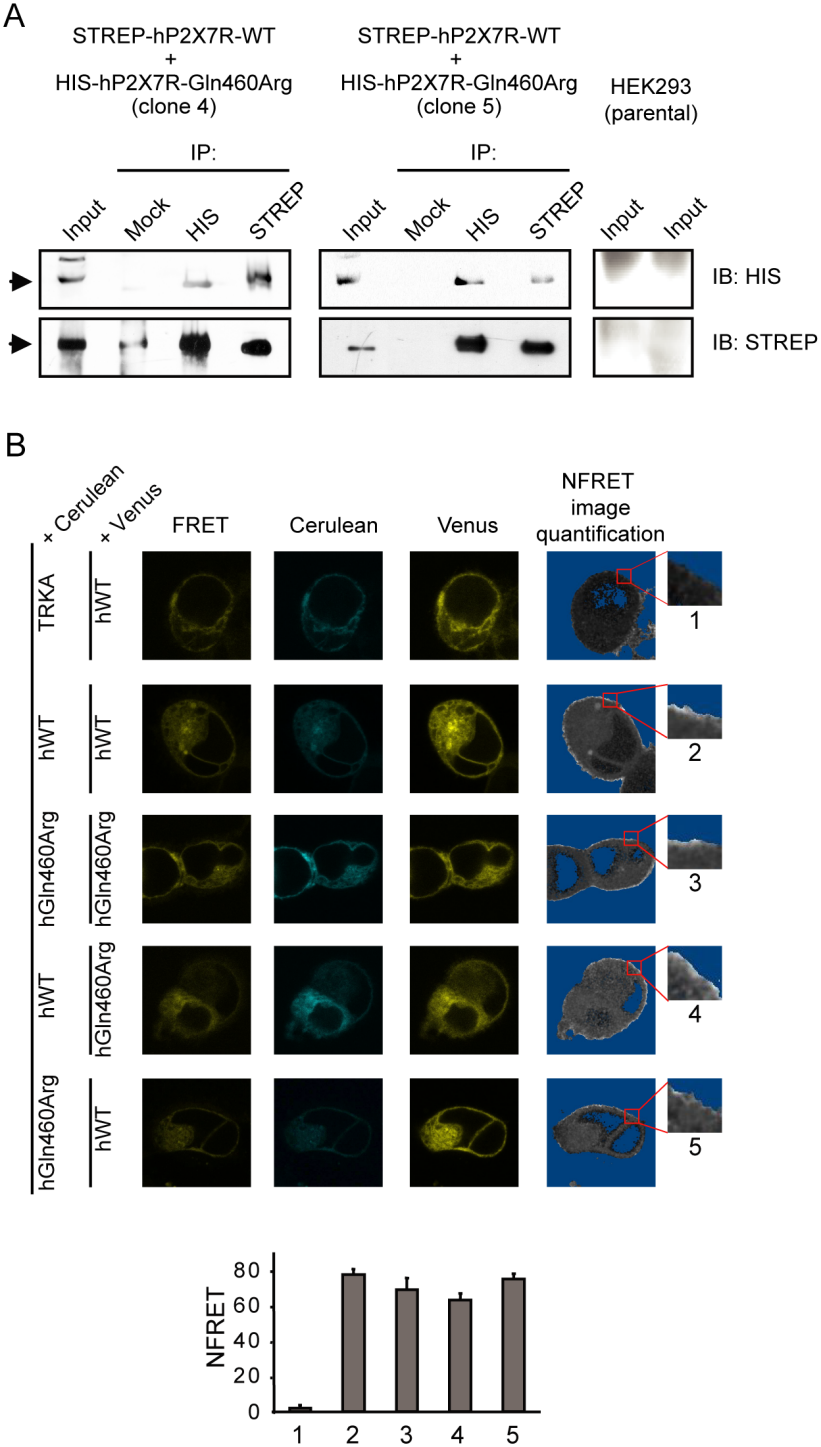
hP2X7R-Gln460Arg interacts with hP2X7R-WT at the cell membrane. (A) Immunoprecipitation (IP) assays on HEK293 clones constitutively co-expressing STREP-tagged hP2X7R-WT and HIS-tagged hP2X7R-Gln460Arg, using anti-HIS (αHIS) and anti-STREP (αSTREP) antibodies for the immunoblotting (IB). One representative experiment out of four with similar results is shown from two out of four clones analyzed. Mock: IP performed in parallel with normal mouse IgGs. Right: Control panel showing that neither HIS-tagged hP2X7R-Gln460Arg nor STREP-tagged hP2X7R-WT were detected in untransfected HEK293 cells. Duplicates are shown. (B) FRET-based confirmation of interaction between hP2X7R-Gln460Arg with hP2X7R-WT. Pixel-by-pixel quantification of sensitized emission FRET on living cells. First column: FRET image: Ex 458 nm/Em 530–630 nm. Second and third columns: Cerulean and cp49Venus fluorescence. The fourth column displays the NFRET image of the same cell. Brighter pixels show higher NFRET levels. Pixels with signal amplitude below threshold are shaded blue. Representative cell images for each condition are shown. Insets: representative magnifications of membrane areas where quantifications were performed. FRET Quantification: Measurement of FRET levels in the cell membrane. Each bar represents the mean ± s.e.m. of 5–10 cells, delimiting 4–6 ROIs per cell at the membrane level, in four independent experiments.

P2X7 receptors predominantly form homotrimeric complexes [6]. To analyze whether the observed phenotype could result from direct physical interaction of P2X7R-WT and P2X7R-Gln460Arg subunits, we generated HEK293 clones that stably co-express both P2X7R variants labeled with different tags—hP2X7R-WT with a streptavidin tag (STREP-hP2X7R-WT) and hP2X7R-Gln460Arg with a histidine tag (HIS-hP2X7R-Gln460Arg) (Fig 2A, input lanes). The tagged variants were functionally similar to non-tagged variants in terms of calcium intake (S2 Fig). The interaction of P2X7R-WT with P2X7R-Gln460Arg was demonstrated by co-immunoprecipitation (Fig 2A). IP assays between P2X7R-WT and P2X7R-Gln460Arg were performed using membrane fractions under high stringency conditions (IP buffer with 0.1% SDS). The fact that this interaction is maintained with the addition of the ionic detergent further supports the notion that the interaction in the membrane fraction between both subunits is strong. This result provides evidence for a physical interaction of P2X7R-WT and P2X7R-Gln460Arg subunits.

To further confirm the results of the IPs and to establish whether the underlying interaction occurs at the cell membrane we used fluorescence resonance energy transfer (FRET). To this purpose we fused monomeric variants of Cerulean and Venus fluorescent proteins to the C-terminus of both P2X7R-WT and P2X7R-Gln460Arg, since it has been demonstrated that P2X7R tagged on their C-termini with either CFP or YFP retain functional properties comparable to their wild-type counterpart [45]. As a positive control we co-transfected hP2X7R-WT-Cerulean and hP2X7R-WT-cp49Venus or hP2X7R-Gln460Arg-Cerulean and hP2X7R-Gln460Arg-cp49Venus. The co-expression yielded high levels of FRET signal due to the expected interaction between Cerulean and cp49Venus fluorescent proteins within the P2X7R trimer (Fig 2B). In contrast, P2X7R did not interact with tyrosine kinase receptor A (TRKA), which served as a negative control. When hP2X7R-WT and hP2X7R-Gln460Arg were co-expressed in the same cell, FRET levels similar to the positive controls were obtained, irrespective of which fluorophore was attached to the receptor variants. This supports not only a direct interaction between hP2X7R-WT and hP2X7R-Gln460Arg but also localizes the interaction to the cell membrane trimer (Fig 2B).

### Rescue of heterozygous phenotype via base pair-specific silencing of P2X7R-WT and P2X7R-Gln460Arg

**Fig 3:**
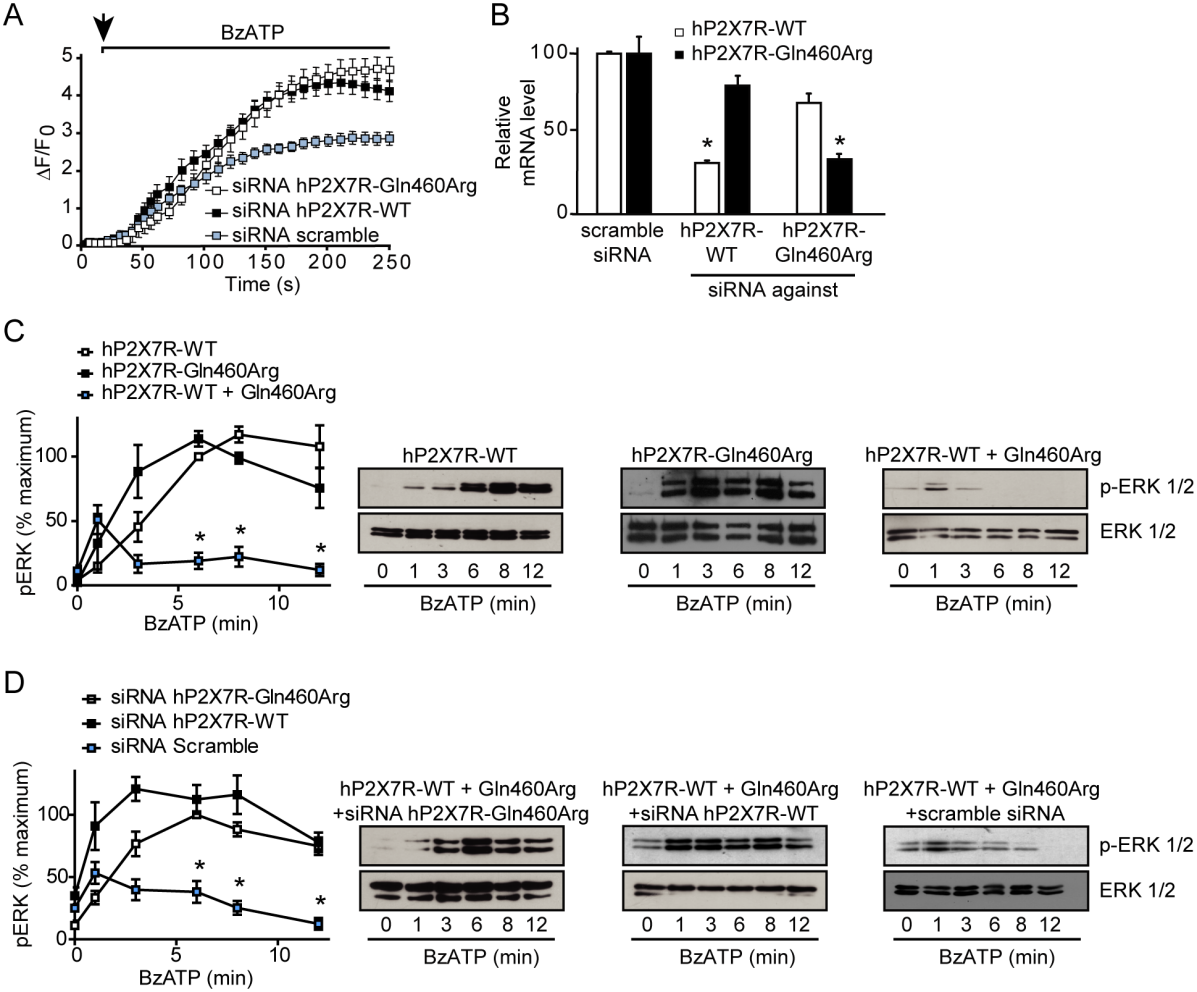
Silencing of either subunit in cells co-expressing hP2X7R-Gln460Arg and hP2X7R-WT restores normal P2X7R function. (A) Increase of intracellular calcium triggered by BzATP (50 μM) was evaluated in hP2X7R-WT and hP2X7R-Gln460Arg co-expressing HEK293 cells transfected with the corresponding scramble siRNA control, WT- or Gln460Arg-specific P2X7R siRNAs (100 nM) for 72 h (repeated measures ANOVA, *P* < 0.001 siRNA hP2X7R-WT and siRNA hP2X7R-Gln460Arg versus scramble siRNA; n = 4). (B) Silencing of mRNA coding for P2X7R-WT or P2X7R-Gln460Arg was confirmed by quantitative real-time RT-PCR (One-way ANOVA, *P < 0.01 versus scramble siRNA; n = 3). (C) BzATP (50 μM)-induced activation of p-ERK 1/2 in HEK293 cells expressing hP2X7R-WT (left), hP2X7R-Gln460Arg (middle) and co-expressing hP2X7R-WT and hP2X7R-Gln460Arg (right). Each value of pERK1/2 was normalized to total ERK1/2. Results are expressed as the percentage of pERK1/2 obtained at 6 minutes of stimulation in hP2X7R-WT cells ± s.e.m. from 3 independent experiments. One-way ANOVA, * P < 0.05 versus hP2X7R-WT and versus hP2X7R-Gln460Arg at the same time points. WBs from a representative experiment are shown. (D) HEK293 cells co-expressing hP2X7R-WT and hP2X7R-Gln460Arg P2X7R variants were silenced using siRNAs that specifically target hP2X7R-Gln460Arg (left), hP2X7R-WT (middle) and with the corresponding scramble siRNA as a control (right). Each value of pERK1/2 was normalized to total ERK1/2. Results are expressed as the percentage of pERK1/2 obtained at 6 minutes of stimulation in siRNA hP2X7R-Gln460Arg cells ± s.e.m. from 3 independent experiments. One-way ANOVA, * P < 0.05 versus siRNA hP2X7R-WT and versus siRNA hP2X7R-Gln460Arg at the same time points. WBs from a representative experiment are shown.

To further confirm that the co-expression of both types of subunits leads to a diminished receptor function, we used the siRNA approach directed to silence each of the single base variants. We designed two pair of siRNA oligos, each one of them specifically silencing either P2X7R-WT or P2X7R-Gln460Arg variant, but not the other (S3 Fig). Thus, we assessed calcium intake and ERK 1/2 phosphorylation on HEK293 cells co-expressing P2X7R-WT and P2X7R-Gln460Arg transfected with siRNAs specifically targeting either the WT or Gln460Arg P2X7R variant. As shown in Fig 3A, the blunted calcium response of stable double clones (in this case transfected with scramble siRNA as a control) was recovered when either P2X7R-WT or P2X7R-Gln460Arg channels were specifically silenced. In both cases, the resulting maximal activation resembles that seen in clones expressing only one type of receptor, P2X7R-WT or P2X7R-Gln460Arg. As expected, a slight change in the kinetics as compared to Fig 1 is observed in the presence of siRNA due to the transient transfection conditions.

Quantitative real-time PCR was used to check the specificity of the silencing experiment at the transcriptional level. As shown in Fig 3B, cells transfected with siRNAs specifically targeting P2X7R-WT had lower levels of mRNA coding for P2X7R-WT, while P2X7R-Gln460Arg transcript levels remained almost unaffected. Transfection of siRNAs specifically targeting P2X7R-Gln460Arg resulted in unchanged levels of mRNA coding for P2X7R-WT and reduction of mRNA encoding P2X7R-Gln460Arg.

Upon transfection of stably co-expressing HEK293 cells with siRNAs knocking down either hP2X7R-WT or hP2X7R-Gln460Arg pERK 1/2 activation was re-established, resembling that of clones expressing single P2X7R variants, which does not occur when transfecting scramble siRNA (Fig 3C). After silencing P2X7R-Gln460Arg, the pERK 1/2 profile turned out to be very similar to that seen on HEK293 cells expressing only P2X7R-WT. Accordingly, silencing of P2X7R-WT showed an ERK 1/2 activation curve similar to the one of P2X7R-Gln460Arg HEK293 cells.

## Discussion

The present study provides direct evidence that the co-expression of P2X7R-WT with the P2X7R-Gln460Arg polymorphic variant causes a significant reduction of normal receptor function.

The impact of Gln460Arg amino acid substitution on P2X7R signal transduction might be related to the binding of the C-terminal P2X7R domain to one of the numerous intracellular signaling components. For instance a Src homology 3 (SH3, residues 441–460) protein binding domain, which encompasses the Gln460Arg polymorphism has been identified [46]. Interaction of a Src tyrosine kinase with this SH3 binding domain ultimately leads to ERK 1/2 phosphorylation [9]. Hence, hetero-oligomerization with P2X7R-Gln460Arg may change the conformation of the domain involved in the interaction with Src tyrosine kinases, leading to an altered, i.e. reduced, ERK 1/2 signal transduction. This view is in line with data showing that the truncated P2X7R lacking the C-terminal domain is able to form heteromers with P2X7R-WT that show a blunted activation of downstream events [47].

Hetero-oligomerization of P2X7R, either with splice variants [47] or natural mutations such as described in this paper, may be a general mechanism for regulation of P2X7R and other ion channels as well. The IP and FRET experiments suggest that the Gln460Arg polymorphism does not impair oligomerization between the wild-type and Gln460Arg variant of the P2X7R. The oligomerization has been related to the cysteine residues located in the extracellular loop of P2X7R subunits that form inter-subunit disulfide bonds [48], which are not affected by the polymorphism.

Based on the function of P2X7R and its association with human diseases, P2X7R has been proposed as a potential therapeutic target for disorders of the nervous system such as inflammatory and neuropathic pain, stroke, spinal cord injury, Alzheimer's disease, multiple sclerosis, major depression and bipolar disorder [49–52]. For example, inflammation [53–55] and glial cell function [56, 57] have been implicated in the pathogenesis of depression. The fact that changes in immune mediators such as pro-inflammatory cytokines are repeatedly observed in patients with mood disorders further supports a potential role of P2X7R in disease etiology [58–60]. The loss of function of P2X7R due to heteromerization with Gln460Arg P2X7R may impact glial cells of patients with affective disorder carrying this mutation. Studies on heterozygous animal models and human patients necessarily should be conducted in order to challenge this mechanism. It will be interesting to verify if functions such as cytokine production or inflammatory actions of P2X7R are, as expected, affected by the P2X7R-WT:P2X7R-Gln460Arg hetero-oligomerization mechanism.

Hetero-oligomerization of P2X7R, either with splice or polymorphic variants may represent a general mechanism for regulation of P2X7Rs and of other ion channels. However, the mechanism identified for the Gln460Arg polymorphism seems to be unique, since direct loss-of-function has been described for many other P2X7R variants. Interestingly, P2X7R outnumbers all other P2X receptor family members with respect to the frequency of non-synonymous SNPs [61]. This high number of polymorphisms might to some extent reflect evolutionary adaptation related to the role of P2X7R in modulating innate immune function [14]. We provide a description of the function of the Gln460Arg SNP showing at the molecular level that this polymorphism results in a loss of function only when interaction occurs between mutated and normal subunits. Furthermore, structural insights are required to mechanistically understand the functional consequences of interaction between hP2X7R-WT and hP2X7R-Gln460Arg subunits and its implication in mood disorders.

## Supporting Information

S1 Fig Analysis of P2X expression in HEK293 cells. (A) Quantification of mRNA expression of P2X family members and exogenously expressed hP2X7R variants in HEK293 cells that endogenously do not express P2X7R. RPL19 was used as a housekeeping gene for normalization. (B) Expression of hP2X7R in parental and stable HEK293 cells on mRNA and protein level. Expression of human hP2X7R-WT and hP2X7R-Gln460Arg mRNA was demonstrated by RT-PCR and subsequent restriction digest of the 557-bp RT-PCR product with PvuII resulting in a 171 bp, 332 bp and 54 bp fragment for the human wild-type and a 503-bp and 54-bp fragment for the human mutant construct (the SNP in the P2X7R-Gln460Arg variant leads to loss of one PvuII restriction site). (C) Protein expression is indicated by WB detection of hP2X7R variants.
(TIF)

S2 Fig Calcium response in tagged P2X7R HEK293 clones. Increase of intracellular calcium triggered by BzATP (50 μM) was evaluated in hP2X7R-WT and hPX7R-Gln460Arg (black circles) and in STREP-hP2X7R-WT and HIS-hP2X7R-Gln460Arg tagged (black squares) co-expressing HEK293 cells. For each cell line, four individual clones were analyzed. Repeated measures ANOVA, non-significant; n = 2.
(TIF)

S3 Fig Silencing specificity of siRNAs against hP2X7R-WT and hP2X7R-Gln460Arg. Increase of intracellular calcium triggered by BzATP (50 μM) was evaluated in (A) hP2X7R-WT and (B) hPX7R-Gln460Arg expressing HEK293 cells transfected either with scramble siRNA control, WT- or Gln460Arg-specific P2X7R siRNAs (100 nM) for 72 h (repeated measures ANOVA, siRNA P2X7-WT *P* < 0.0001 and siRNA P2X7-Gln460Arg non-significant versus scramble siRNA (A); siRNA P2X7-Gln460Arg *P* < 0.0001 and siRNA P2X7-WT non-significant versus scramble siRNA (B); n = 3).
(TIF)
