# Supplementary figures and images for: Co-Expression of Wild-Type P2X7R with Gln460Arg Variant Alters Receptor Function
Source: PLoS One. 2016 Mar 17;11(3):e0151862. doi: 10.1371/journal.pone.0151862 (PMC4795689; doi:10.1371/journal.pone.0151862)

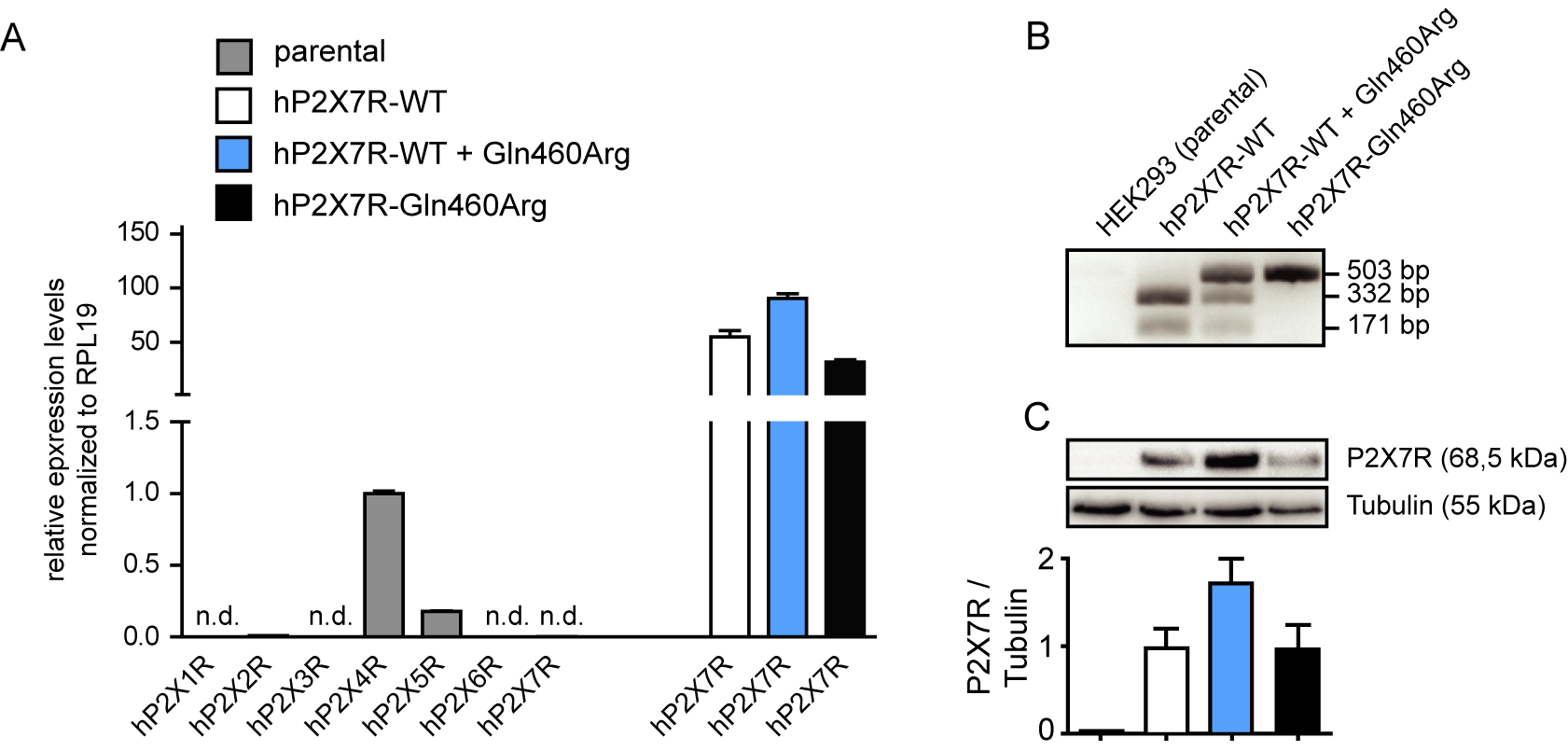

Supplement: S1 Fig — (A) Quantification of mRNA expression of P2X family members and exogenously expressed hP2X7R variants in HEK293 cells that endogenously do not express P2X7R. RPL19 was used as a housekeeping gene for normalization. (B) Expression of hP2X7R in parental and stable HEK293 cells on mRNA and protein level. Expression of human hP2X7R-WT and hP2X7R-Gln460Arg mRNA was demonstrated by RT-PCR and subsequent restriction digest of the 557-bp RT-PCR product with PvuII resulting in a 171 bp, 332 bp and 54 bp fragment for the human wild-type and a 503-bp and 54-bp fragment for the human mutant construct (the SNP in the P2X7R-Gln460Arg variant leads to loss of one PvuII restriction site). (C) Protein expression is indicated by WB detection of hP2X7R variants. (TIF) [file pone.0151862.s001.tif]

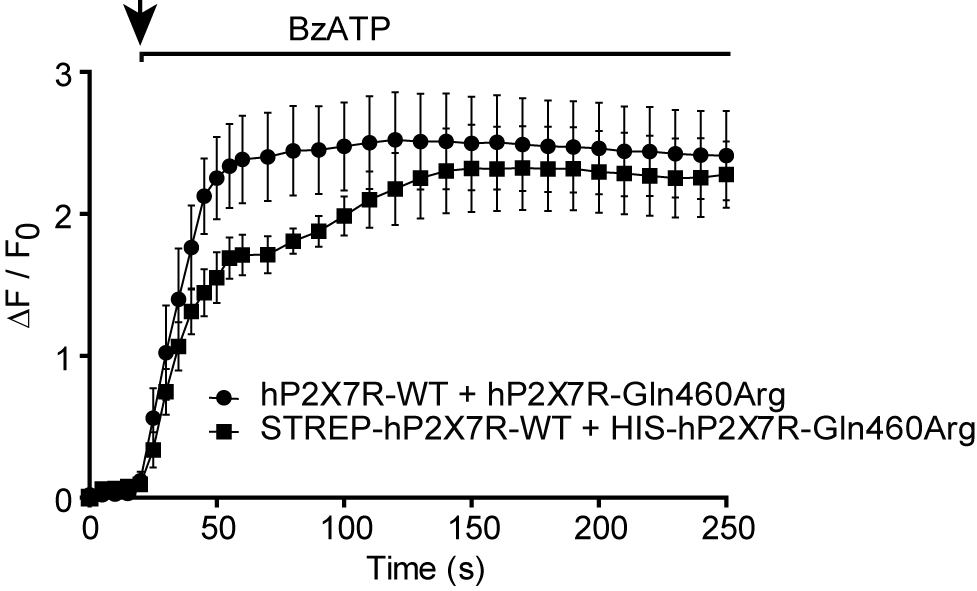

Supplement: S2 Fig — Increase of intracellular calcium triggered by BzATP (50 μM) was evaluated in hP2X7R-WT and hPX7R-Gln460Arg (black circles) and in STREP-hP2X7R-WT and HIS-hP2X7R-Gln460Arg tagged (black squares) co-expressing HEK293 cells. For each cell line, four individual clones were analyzed. Repeated measures ANOVA, non-significant; n = 2. (TIF) [file pone.0151862.s002.tif]

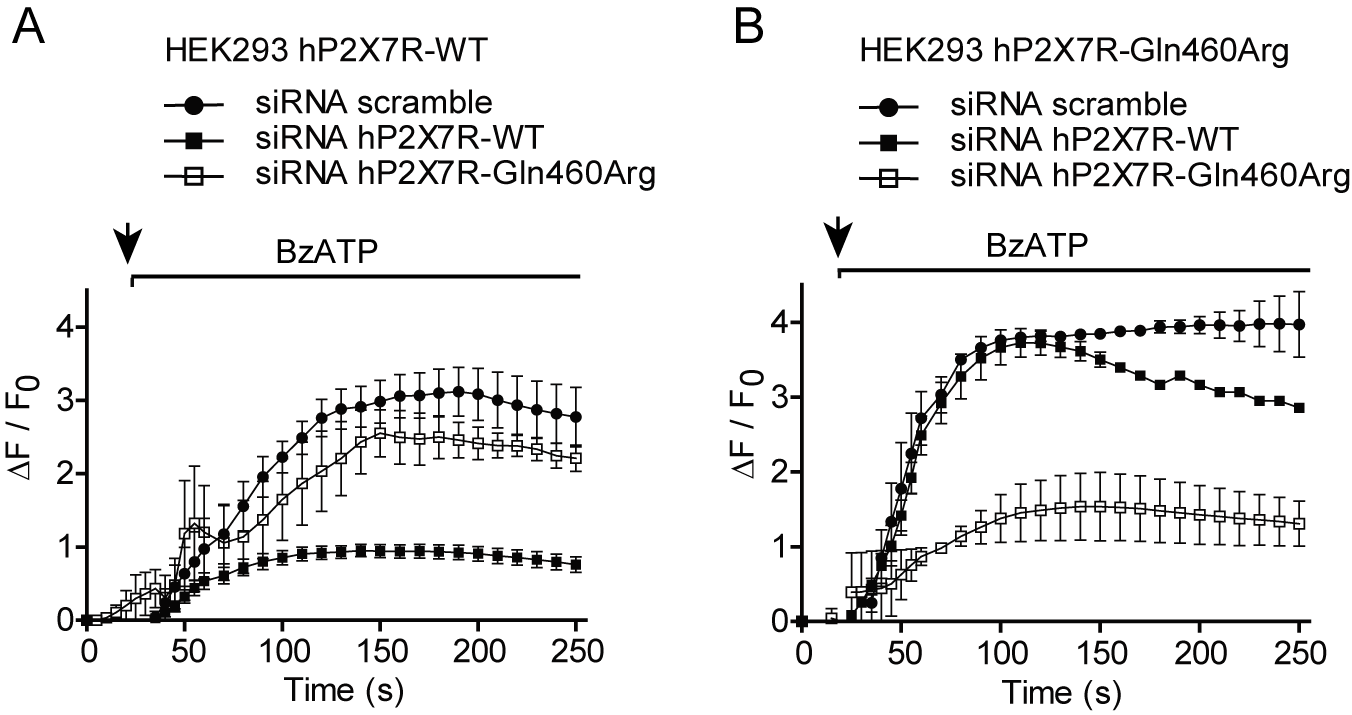

Supplement: S3 Fig — Increase of intracellular calcium triggered by BzATP (50 μM) was evaluated in (A) hP2X7R-WT and (B) hPX7R-Gln460Arg expressing HEK293 cells transfected either with scramble siRNA control, WT- or Gln460Arg-specific P2X7R siRNAs (100 nM) for 72 h (repeated measures ANOVA, siRNA P2X7-WT P < 0.0001 and siRNA P2X7-Gln460Arg non-significant versus scramble siRNA (A); siRNA P2X7-Gln460Arg P < 0.0001 and siRNA P2X7-WT non-significant versus scramble siRNA (B); n = 3). (TIF) [file pone.0151862.s003.tif]
